# Passive immunization of macaques with polyclonal anti-SHIV IgG against a heterologous tier 2 SHIV: outcome depends on IgG dose

**DOI:** 10.1186/1742-4690-11-8

**Published:** 2014-01-20

**Authors:** Anton M Sholukh, Siddappa N Byrareddy, Vivekanandan Shanmuganathan, Girish Hemashettar, Samir K Lakhashe, Robert A Rasmussen, Jennifer D Watkins, Hemant K Vyas, Swati Thorat, Tania Brandstoetter, Muhammad M Mukhtar, John K Yoon, Francis J Novembre, Francois Villinger, Gary Landucci, Donald N Forthal, Sarah Ratcliffe, Iskra Tuero, Marjorie Robert-Guroff, Victoria R Polonis, Miroslawa Bilska, David C Montefiori, Welkin E Johnson, Hildegund C Ertl, Ruth M Ruprecht

**Affiliations:** 1 Department of Virology and Immunology, Texas Biomedical Research Institute, PO Box 760549, San Antonio, TX 78245-0549, USA; 2 Dana-Farber Cancer Institute, Boston, MA, USA; 3 Harvard Medical School, Boston, MA, USA; 4 Yerkes National Primate Research Center, Emory University, Atlanta, GA, USA; 5 Department of Microbiology and Immunology and Laboratory Medicine, Emory University, Atlanta, GA, USA; 6 Department of Pathology and Laboratory Medicine, Emory University, Atlanta, GA, USA; 7 Division of Infectious Diseases, Department of Medicine, University of California, Irvine School of Medicine, Irvine, CA, USA; 8 Department of Biostatistics and Epidemiology, Perelman School of Medicine, University of Pennsylvania, Philadelphia, PA, USA; 9 National Cancer Institute, Center for Cancer Research, Vaccine Branch, Bethesda, MD, USA; 10 The Military HIV Research Program, Walter Reed Army Institute of Research, Silver Spring, MD, USA; 11 Department of Surgery, Duke University School of Medicine, Durham, NC, USA; 12 Department of Biology, Boston College, Boston, MA, USA; 13 The Wistar Institute, Philadelphia, Pennsylvania, USA

**Keywords:** Macaque model, Heterologous R5 SHIV clade C challenge, SHIVIG, Passive immunization, Enhancement of infection, Non-human primate model

## Abstract

**Background:**

A key goal for HIV-1 envelope immunogen design is the induction of cross-reactive neutralizing antibodies (nAbs). As AIDS vaccine recipients will not be exposed to strains exactly matching any immunogens due to multiple HIV-1 quasispecies circulating in the human population worldwide, heterologous SHIV challenges are essential for realistic vaccine efficacy testing in primates. We assessed whether polyclonal IgG, isolated from rhesus monkeys (RMs) with high-titer nAbs (termed SHIVIG), could protect RMs against the R5-tropic tier-2 SHIV-2873Nip, which was heterologous to the viruses or HIV-1 envelopes that had elicited SHIVIG.

**Results:**

SHIVIG demonstrated binding to HIV Gag, Tat, and Env of different clades and competed with the broadly neutralizing antibodies b12, VRC01, 4E10, and 17b. SHIVIG neutralized tier 1 and tier 2 viruses, including SHIV-2873Nip. NK-cell depletion decreased the neutralizing activity of SHIVIG 20-fold in PBMC assays. Although SHIVIG neutralized SHIV-2873Nip in vitro, this polyclonal IgG preparation failed to prevent acquisition after repeated intrarectal low-dose virus challenges, but at a dose of 400 mg/kg, it significantly lowered peak viremia (*P* = 0.001). Unexpectedly, single-genome analysis revealed a higher number of transmitted variants at the low dose of 25 mg/kg, implying increased acquisition at low SHIVIG levels. In vitro, SHIVIG demonstrated complement-mediated Ab-dependent enhancement of infection (C’-ADE) at concentrations similar to those observed in plasmas of RMs treated with 25 mg/kg of SHIVIG.

**Conclusion:**

Our primate model data suggest a dual role for polyclonal anti-HIV-1 Abs depending on plasma levels upon virus encounter.

## Background

Although antibody (Ab)-mediated immunity against HIV-1 has been the focus of intense research, the role of Abs in preventing HIV-1 infection remains to be fully elucidated. The RV144 vaccine trial showed moderate efficacy (31.2%) and raised the possibility that non-neutralizing Abs may be associated with protection against HIV-1 acquisition [1]. In this regard, a biologically relevant non-human primate model, in which macaques are passively immunized with Abs against simian-human immunodeficiency virus (SHIV) followed by repeated mucosal challenges with a heterologous R5 SHIV at low doses, may mimic key aspects of HIV-1 transmission among humans and thus play an important role in dissecting the mechanism(s) of Ab action in HIV-1 transmission and its prevention [2]. Another consideration in modeling natural infection in humans is the nature of transmitted HIV-1 strains: most such viruses are relatively difficult to neutralize (tier 2 neutralization phenotype).

Early attempts to use primates to elucidate the role of Abs were conducted with polyclonal IgG isolated from HIV-1-infected subjects (termed HIVIG) and showed either no or complete protection against laboratory-adapted, neutralization-sensitive (tier 1) viruses in chimpanzees depending on challenge virus doses [3-5]. Anti-HIV-1 envelope (Env) Abs have also been evaluated in rhesus monkeys (RMs) challenged with SHIVs encoding HIV-1 *env*, *tat*, *rev* and *vpu*. SHIVs allow assessing the protective role of broadly neutralizing anti-HIV-1 monoclonal Abs (bnmAbs) by passive immunization [reviewed in [2]]. Of note, SHIVs used earlier, such as SHIV_89.6P_, were either X4- or dual-tropic and irreversibly destroyed naïve and memory CD4^+^ T cells within two weeks [6]. When HIVIG alone or combined with bnmAbs was tested against SHIV_89.6PD_[7,8], only the combination of HIVIG with bnmAbs resulted in moderate prevention of virus acquisition. In another pilot study, polyclonal IgG isolated from HIV-1-infected chimpanzees was administered to macaques that were challenged with homologous SHIV. Only two out of 10 macaques remained aviremic, one treated with high-dose IgG and the other one challenged with a low dose of virus [9].

Early passive immunization studies in primates were performed with single high-dose, intravenous virus challenges rather than low-dose mucosal virus exposures that more closely resemble sexual HIV-1 transmission among humans. Recently, a passive immunization study using a combination of polyclonal IgG and bnmAbs was performed against repeated low-dose challenges with the tier 2 R5-tropic SHIV_SF162P3_; virus acquisition could not be prevented even though the challenge virus was homologous to the polyclonal IgG given [10].

We have constructed SHIV-2873Nip, an R5-tropic SHIV carrying a HIV-1 clade C (HIV-C) *env* isolated from a Zambian infant who had rapid disease progression and died within 1 year of birth [11]. SHIV-2873Nip, a tier 2 virus, causes AIDS in RMs with clinical parameters and a disease progression rate similar to those in humans (unpublished data). Thus, the RM/SHIV-2873Nip model is a biologically relevant system to assess the role of Abs in providing protection against lentiviral acquisition.

Here we report passive immunization with SHIVIG, a polyclonal preparation of IgG isolated from RMs chronically infected with clade C SHIV strains carrying envelopes phylogenetically distinct from that of the challenge virus. We tested whether SHIVIG could protect RMs against multiple low-dose intrarectal (i.r.) challenges with SHIV-2873Nip that is heterologous to any viruses or envelopes against which the IgG responses had been elicited. We elected to perform upfront heterologous SHIV challenges to mimic the situation of human AIDS vaccine recipients, who are not likely to be exposed to HIV-1 strains that exactly match the composition of the immunogen(s). Thus, our passive immunization study in the primate model was designed to assess the level of cross-neutralizing IgG needed for in vivo protection; such information would be helpful to guide future development of Ab-based immunogens. Unexpectedly, virus acquisition was not prevented at any SHIVIG dose. Rather, we found evidence of partial inhibition of acute viremia or increased virus acquisition, depending on the SHIVIG dose.

## Results

### Selection of RM donors and isolation of total IgG

We selected RMs with high neutralizing antibody (nAb) titers against several viruses (Table 1). We had used these animals in previous virus adaptations, titrations, and vaccine studies [12-14]. All animals were chronically infected with SHIV-1157ip [12] and/or SHIV-1157ipd3N4 [15]; some monkeys had also been vaccinated (Additional file 1: Table S1) or challenged with *Schistosoma mansoni*, which the animals cleared after the acute stage of parasite infection [13]. Four RMs were long-term non-progressors. Some RMs developed Abs that neutralized heterologous viruses at high titers (Table 1). Sera collected sequentially from such SHIV-infected RMs were used to isolate total IgG; the final preparation was named SHIVIG (Methods).

**Table 1:** Neutralizing antibody titers of rhesus monkeys selected as SHIVIG donors

**RM**	**Homologous clade C viruses**		**Heterologous clade C viruses**						**Heterologous clade B viruses**			
	**SHIV-1157ip (early, tier 1)**	**SHIV-1157ipd3N4 (late, tier 2)**	**HIV**_**ZM135M**_	**HIV**_**1084i**_	**HIV**_**pIndieC**_	**HIV**_**ZM233M.PB6**_	**HIV**_**ZM109F**_	**SHIV-2873Nip**	**SHIV**_**SF162P3**_	**HIV**_**SF162.LS**_	**HIV**_**NL4-3**_	**SHIV**_**89.6P**_
RAo-8	2,048	>640	<20		128	42	<20	>640	<40	220	32	
RCt-10	360	>640						>640		6,500		
RHo-10	250	>640						>640				
RHy-9	600	>10,240	<20	100	90	<20	<20	>640		173		100
RJa-9	1,800	>10,240	<20		128	59	22	2,048	68	35,770	128	
RLu-9	>1,280	>640	<20	50	47	<20	<20	>640		58		75
RMf-9	2,048	>640	<20		128	<20	35	>640	78	18,303	128	
RPo-10	200	>640						>640				
RTs-7	2,048	>640			128			>640			32	

NAb titers (reciprocal dilution of sera giving 50% reduction in virus replication) were determined in TZM-bl cells or, for SHIV-1157ipd3N4 and SHIV-2873Nip, in human PBMC-based assays. The SHIVs were prepared in RM PBMC; HIV_1084i_, HIV_NL4-3_ and HIV_pIndieC_ were prepared in human PBMC; nAb titers against the remaining HIV strains were performed using pseudoviruses generated in transfected 293 T cells. All neutralization assays were performed at least in duplicate.

### SHIVIG binding specificity and in vitro neutralization potency

**Figure 1:**
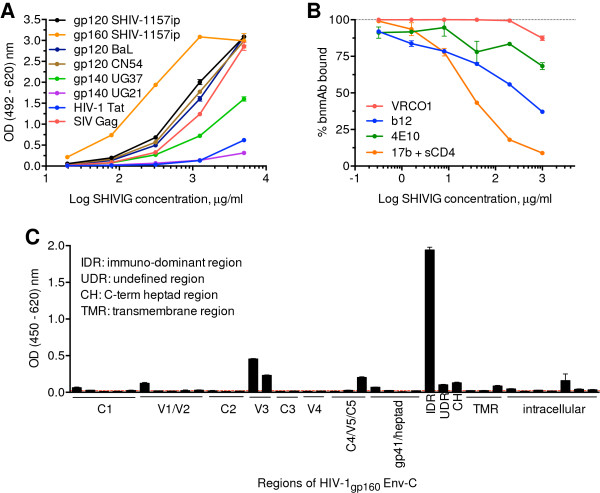
**SHIVIG characterization. A.** SHIVIG binding to soluble HIV and SIV proteins was tested by ELISA. Proteins were captured on the plates and probed with serial dilutions of SHIVIG. Binding was detected as described in Methods. Each data point represents the mean ± SEM (n = 3). Env proteins were derived from the following HIV or SHIV strains: clade A, UG37; B, BaL; C, CN54 and SHIV-1157ip; D, UG21. **B.** Competitive ELISA with the CD4-binding site-specific bnmAbs b12 and VRC01; gp41 MPER-specific bnmAb 4E10; and CD4-inducible site-specific bnmAb 17b. Plates were coated with HIV-1_CN54_ gp120 for b12 and VRC01 analysis or SHIV-1157i gp160 for 4E10 and further incubated with 0.5 μg/ml of the bnmAb indicated along with different concentrations of SHIVIG in duplicates or triplicates. For 17b mAb, HIV-1_CN54_ gp120 was captured by polyclonal sheep anti-gp120 Abs coated on the plate and the assay was continued as described in Methods. The y-axis indicates OD percentage of maximal binding, which is determined as the reading without SHIVIG (100% binding is marked by dashed line). The irrelevant mAb Fm-6 (anti-SARS virus) and naïve RM IgG were used as negative controls (not depicted). **C.** ELISA of SHIVIG with consensus clade C Env peptides. The x-axis designates pools of peptides assigned to HIV-1 Env regions. Dotted line represents background. IDR, immunodominant region; UDR, undefined region in gp41; CH, C-terminal heptad region; TMR, transmembrane region. Each data point represents the mean ± SEM (n = 3). All experiments were repeated at least twice.

By ELISA, SHIVIG cross-recognized HIV-1 gp120 and gp140 of clades A, B and C, but did not bind to clade D Env. Anti-Gag binding activity was comparable to that against Env whereas level of Abs against HIV-1 Tat was low (Figure 1A). Competition ELISA with bnmAbs b12, VRC01, 4E10 and 17b demonstrated the presence of Abs directed against the CD4-binding site, the CD4-inducible site and the membrane-proximal external region (MPER) of gp41, respectively (Figure 1B). Detailed analysis of anti-Env binding with consensus clade C gp120 peptides revealed Abs reacting with V1 and V3 and the C5 region (Figure 1C). Within gp41, SHIVIG interacted with the immunodominant region (IDR), the C-terminal heptad region (CH), an undefined region between IDR and CH as well as with the intracellular portion. Thus, SHIVIG demonstrated antigen specificity similar to plasma samples from HIV-1-infected subjects [16].

Next, SHIVIG was tested for neutralization potency against tier 1 and tier 2 viruses (Table 2). While SHIVIG efficiently neutralized all three tier 1 viruses, neutralization of tier 2 strains was less potent: only two out of seven such viruses tested, including SHIV-2873Nip, were neutralized at 60% efficiency. To establish the neutralization potency of SHIVIG against the intended heterologous challenge SHIV-2873Nip in a more relevant assay, we performed human peripheral blood mononuclear cell (PBMC)-based assays with cells obtained from several donors. Neutralization of SHIV-2873Nip was as high as 95% (1 mg/ml SHIVIG). The half maximal inhibitory concentration (IC_50_) values ranged from 0.2 to 144 μg/ml (mean, 11.9 μg/ml) depending on the donor, a variability that is likely due to the inherent heterogeneity of PBMC from different donors, including natural killer (NK) cell receptor polymorphism [17].

**Table 2:** Percent neutralization of different virus strains by SHIVIG in TZM-bl cell-based assay

**Virus**	**Clade**	**Tier**	**SHIVIG**	^**b**^**Positive control**
KNH1088.ec5	A	2	0	83
SF162 PV	B	1	94	100
BaL.ec1	B	1	92	99
GS015.ec12	C	1	74	95
GS 014 IMC	C	2	0	82
E0836M4.ec3	D	2	0	80
CM235.ec5	AE	2	66	100
GS 020 IMC	AE	2	0	38
55815.ec3	AG	2	0	75
SHIV-2873Nip	C	2	^a^58	^c^56

SHIVIG was tested at 0.5 mg/ml, except where noted. ^a^Neutralization obtained with 1 mg/ml of SHIVIG. ^b^An HIV-positive serum pool was used as positive control, except as indicated. ^c^BnmAb VRC01 (50 μg/ml) was used as positive control. IMC, infectious molecular clone. All neutralization assays were performed at least in duplicate.

### In vitro SHIVIG effector functions

**Figure 2:**
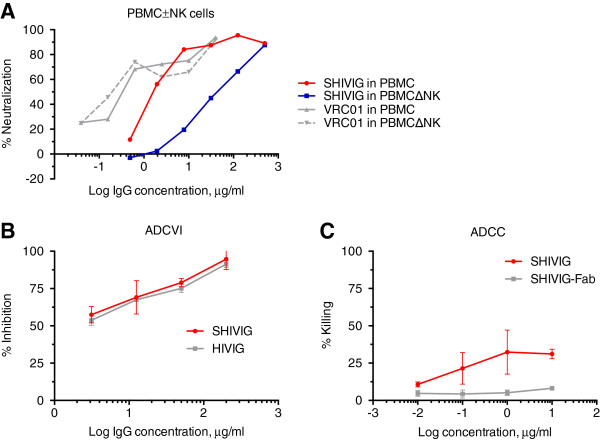
**In vitro neutralization and effector functions of SHIVIG against the challenge virus, SHIV-2873Nip. A.** Neutralizing activity of SHIVIG in PBMC assays with or without NK cells. Serially diluted SHIVIG was assayed with unfractionated human PBMC (red) and PBMC depleted of NK cells (blue) in triplicates. VRC01 was used as a positive control and was analyzed with unfractionated human PBMC (solid grey line) and PBMC depleted of NK cells (dashed grey line) as described in Methods. **B.** ADCVI activity of SHIVIG and HIVIG (IgG from a pool of HIV-positive donors; positive control; squares) was assessed against SHIV-2873Nip as described in Methods. The graph shows the percentage of virus inhibition (y axis) by increasing concentrations of SHIVIG (red) or HIVIG (grey) normalized by values obtained for negative controls at the same concentrations. **C.** ADCC activity of serially diluted SHIVIG (red) and SHIVIG-derived Fab (grey) were tested in triplicates with CEM-NKr target cells coated with gp120 of HIV_96ZM651_ (clade C) and human PBMC as effector cells. The graph shows killing (in percentage) of target cells in the presence of increasing concentrations of SHIVIG or SHIVIG-Fab. Experiments were repeated in triplicate, and representative mean results are shown.

Increasing evidence suggests a role of NK cells in early antiviral defenses, including HIV-1 [18]. We performed PBMC-based neutralization assays with/without NK cells (Figure 2A). The virus/Ab mixture was left with the PBMC until day 4, thus allowing for ADCC activity. NK-cell depletion led to a significant decrease of neutralization potency of SHIVIG, characterized by a 20-fold increase of IC_50_ value from 2.2 μg/ml (PBMC) to 42 μg/ml (PBMC minus NK cells); virus neutralization by VRC01 was not affected.

SHIVIG almost completely inhibited the challenge virus at 0.2 mg/ml in the ADCVI assay performed with CEM.NKr.CCR5 target cells and human PBMC as effector cells (Figure 2B). To measure ADCC (Figure 2C), the same target cells were coated with clade C HIV_96ZM651_ gp120; PBMC were used as effector cells. We could not reach >35% target cell killing, possibly due to imperfect recognition of the heterologous gp120 on the cell surface by SHIVIG.

### Animal study design and SHIVIG pharmacokinetics

**Figure 3:**
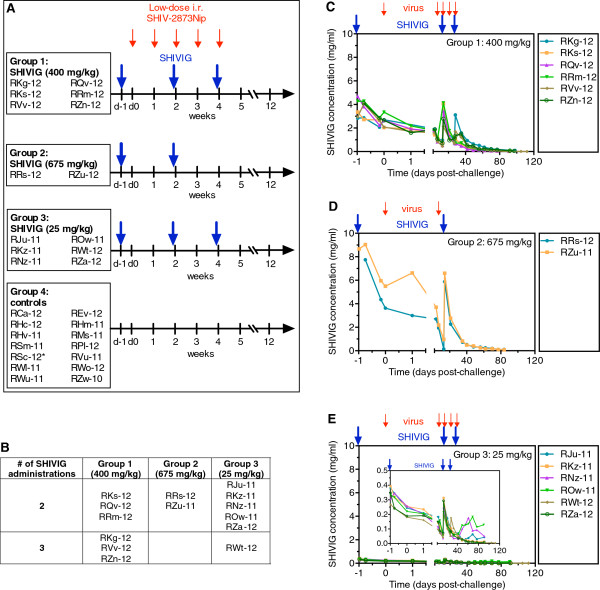
**Study design and SHIVIG pharmacokinetics. A.** Animal study design and timeline. Four groups of female RMs were enrolled. Group 1 (n = 6) received 400 mg/kg of SHIVIG, Group 2 (n = 2), 675 mg/kg, and Group 3 (n = 6), 25 mg/kg, respectively. Group 4 (n = 14) RMs served as virus-only controls. Ten animals were enrolled and four additional animals used for virus titration given the identical virus dose served as additional controls. The challenge virus, SHIV-2873Nip, had been titrated i.r. to yield systemic infection (>10^4^ viral RNA copies) in untreated monkeys after a maximum of 5 weekly challenges (small red arrows); the challenge dose was 5,000 50% tissue culture infectious doses (TCID_50_) measured by TZM-bl assay. SHIVIG infusions (large blue arrows) as well as viral challenges were stopped after the monkeys became viremic (>10^4^ viral RNA copies/ml). **B.** The number of SHIVIG administrations that RMs from different groups received while their vRNA loads were <10^4^ copies/ml. **C-E.** SHIVIG pharmacokinetics in RM groups. Large blue arrows indicate biweekly SHIVIG administrations and small red arrows indicate SHIV-2873Nip challenges; **C.** Group 1 (400 mg/kg of SHIVIG, n = 6). **D.** Group 2 (675 mg/kg of SHIVIG, n = 2); **E.** Group 3 (25 mg/kg of SHIVIG, n = 6). The insert represents the same graph showing the lower Y axis range. Serial dilutions of RM plasmas were incubated on ELISA plates to which HIV-1 BaL gp120 had been added and preimmobilized. Binding was assessed as described in Methods. All samples were assayed in triplicate.

Next, we passively immunized RMs with three different doses of SHIVIG against weekly low-dose i.r. SHIV-2873Nip challenges (Figure 3A). Group 1 RMs received SHIVIG at 400 mg/kg, Group 2 at 675 mg/kg, and Group 3 at 25 mg/kg. Control Group 4 macaques were left untreated. Macaques with different MHC and TRIM5α genotypes were distributed approximately evenly (Additional file 2: Table S2). SHIVIG was administered intravenously every two weeks, 24 h before the first, third and fifth low-dose viral challenges. A maximum of three SHIVIG administrations was given. All RMs were challenged i.r. weekly with low-dose SHIV-2873Nip (Figure 3A). SHIVIG infusions (Figure 3B) as well as viral challenges were stopped once an animal had >10^4^ vRNA copies/ml, a level typically associated with subsequent seroconversion and hence persistent systemic infection.

SHIVIG plasma levels were measured by ELISA with gp120 as antigen (Figure 3C-E). SHIVIG had a biphasic decay with an initial drop during the first 24 h and a mean half-life of 12.2 ± 1.8 days after the last administration. These values are compatible with those observed by others for HIVIG and nAbs administered i.v. to RMs [7]. The mean SHIVIG concentration in RM plasma on the day of first viral challenge was 2.6 ± 0.2 mg/ml for Group 1 (400 mg/kg), 4.6 ± 0.9 mg/ml for Group 2 (675 mg/kg) and 0.22 ± 0.01 mg/ml for Group 3 (25 mg/kg).

The penetration of SHIVIG into mucosal secretions was assessed by measuring anti-gp120 binding on day 13 in vaginal washes. The latter were collected because rectal mucosae could not be manipulated during i.r. virus challenges. In Group 1 RMs, SHIVIG represented 7.6 ± 1.4% of total vaginal IgG (Table 3). SHIVIG concentration in mucosal secretions of Group 3 (25 mg/kg) was below the detection limit.

**Table 3:** Percentage of SHIVIG in total vaginal IgG of Group 1 RMs two weeks after administration

**Monkey**	**% SHIVIG**
RKg-12	12.4
RKs-12	5.2
RQv-12	8.7
RRm-12	4.3
RVv-12	4.5
RZn-12	10.4

The content of SHIVIG and total RM IgG was determined as described in and Methods. All samples were analyzed in triplicates.

### SHIV-2873Nip challenges

**Figure 4:**
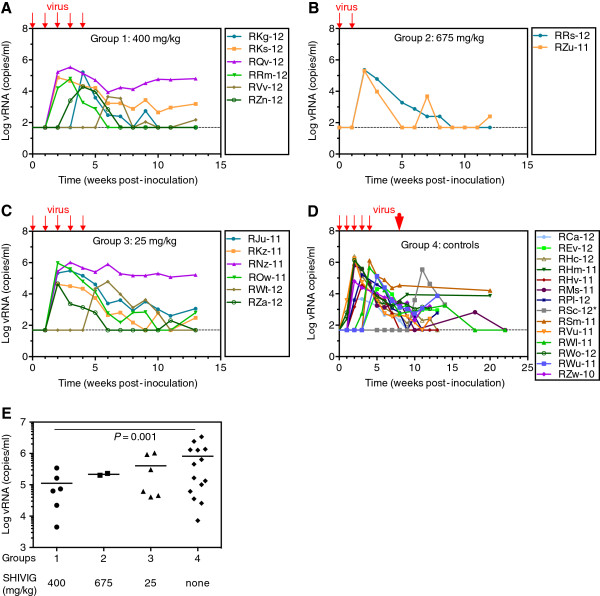
**Plasma viral RNA (vRNA) loads after SHIV-2873Nip challenges. A-D.** Graphs represent vRNA loads after viral challenges for Group 1–4, respectively. Small red arrows indicate 5 low-dose SHIV-2873Nip challenges. Animals in Group 2 **B** became infected after the second viral challenge and further challenges were omitted. **D.** Virus-only controls. Three animals from this group could not be challenged at week 4 (inclement weather forced closure of the primate center) and one RM (*RSc-12) became viremic only after the high-dose challenge (30,000 TCID_50_) at week 7 (large red arrow). During the 2-week interval between the 4^th^ and 5^th^ low-dose challenge, this RM had developed SIV Gag-specific proliferative responses (data not shown). **E.** Statistical analysis of the peak of vRNA loads. The dashed line represents the limit of detection (50 copies/ml). For control Group 4, peak vRNA load was calculated including the animals with protocol breach. Peak viremia levels were compared using negative binomial regression, in order to adequately model the non-normality of the observed peak levels. Raw p-values from pair-wise comparisons with the control group were adjusted using Dunn’s method.

The repeated low-dose challenges resulted in infection of all passively immunized RMs (Figure 4A-D), although SHIVIG neutralized SHIV-2873Nip almost completely in vitro (Figure 2A). Mean time from initial virus exposure to peak viremia was 3.7 ± 1.4 weeks for Group 1, two weeks for Group 2, 3 ± 1.6 weeks for Group 3, and 2.7 ± 0.9 weeks for Group 4. These differences were not statistically significant.

### Higher SHIVIG doses demonstrate partial protection

We performed statistical analysis of parameters for viremia and found no significant differences in the time to viremia, time to peak viremia and area-under-the-curve (AUC) between different groups. However, Group 1 monkeys (400 mg/kg) demonstrated significantly lower peak viral RNA (vRNA) loads compared with control RMs (*P* = 0.001) (Figure 4E), indicating that SHIVIG administered at 400 mg/kg conferred significant partial protection against SHIV-2873Nip. Although the mean peak vRNA load of Group 3 (675 mg/kg) was lower compared with that of the control, statistical significance was not reached because we could only enroll two RMs. Statistical analyses did not reveal any correlation between viremia and MHC or TRIM5α genotypes.

### All RMs seroconverted post challenge

All macaques seroconverted by Western blot with HIV-1/2 proteins and IgM ELISA with SIV Gag and HIV-1 Env as antigens (data not shown). IgM ELISAs were performed to distinguish between SHIVIG-associated titers that decline with time and Ab responses induced by breakthrough infection. Notably, three RMs from Group 3 (RNz-11, ROw-11, and RJu-11) developed rapid IgG responses after day 40 post-challenge and breakthrough infection (insert, Figure 3E). This observation agrees with recent reports [10] and indicates that a suboptimal concentration of virus-specific Abs in plasma at the time of virus challenge might facilitate the generation of humoral immune responses to the challenge virus.

### SHIV-2873Nip is not completely neutralized by plasma samples of passively immunized RMs

Next, we assessed whether challenge virus neutralization by RM plasma after SHIVIG administration was comparable to in vitro assay values obtained for SHIVIG itself. Thus, we measured the neutralizing activity of RM plasma samples from the day of first virus challenge. All measurements were carried out using human PBMC obtained from the same individual. From every experimental group, we selected the animals with the highest and lowest viremia levels to test whether the differences could be linked to SHIVIG. IC_50_ values extrapolated from the plasma samples were consistent with IC_50_ values obtained earlier for the SHIVIG prep (Table 4). None of the plasma samples completely prevented PBMC infection. Furthermore, no significant differences were observed for IC_50_ values and percent neutralization for RMs with high versus low peak vRNA loads. Of note, plasma samples of Group 3 RMs demonstrated a lower percentage of SHIV-2873Nip neutralization than those from animals of Groups 1 and 2. This suggests correlative trends between the administered dose of SHIVIG, neutralization, and partial protection against SHIV-2873Nip.

**Table 4:** Neutralization of SHIV-2873Nip by plasma samples of SHIVIG recipients

**Group (mg/kg of SHIVIG)**	**Monkey**	**Peak vRNA load (copies/ml)**	**Day of 1**^**st **^**challenge**	
			**Highest % of neutralization**	**SHIVIG plasma IC**_**50 **_**(μg/ml)**
Group 1	RVv-12	4,500	90.1	12.9
(400)	RQv-12	342,850	85.7	10.9
Group 2	RZu-11	201,700	74.7 (91.8)	6.0
(675)	RRs-12	229,600	88.8 (95.6)	4.8
Group 3	RWt-12	61,750	58.5	6.2
(25)	RNz-11	1,032,600	66.7	3.3

The highest percent of neutralization was seen at a plasma dilution of 1:6. Values in parentheses represent the highest % of neutralization reached for RZu-11 and RRs-11 at plasma dilutions 1:54 and 1:18, respectively. SHIVIG plasma IC_50_ concentrations were determined using the concentration of SHIVIG in RM plasma on the day of challenge and the dilution of the same plasma sample showing 50% of neutralization in PBMC assay. Neutralization assays were performed at least in triplicate. Measurements of viral loads were performed in duplicate.

### RMs treated with the low SHIVIG dose have more transmitted virus quasispecies

**Figure 5:**
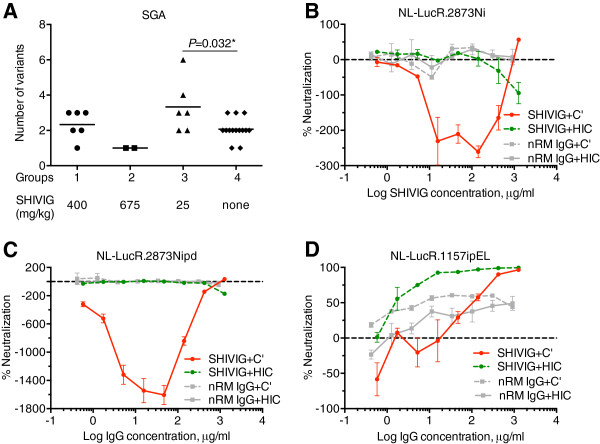
**Single-genome analysis (SGA) and C’-ADE. A.** Number of SHIV-2873Nip quasispecies at peak viremia. The number of quasispecies was analyzed by SGA of the V1/V2 loop region of SHIV-2873Nip *env*. For every RM, at least 10 individual clones were obtained by limiting dilution PCR followed by sequencing. Sequence readout was performed using both strands of DNA. Statistical significance was assessed by Mann–Whitney test with Holm correction (*P* = 0.032). **B-D.** C’-ADE activity of SHIVIG in SupT1.R5 cells tested against different viruses built from the HIV_NL4-3_ backbone and containing the envelope of various SHIV or HIV-C strains as well as a *Renilla* luciferase (LucR) reporter gene. **B.** NL-LucR.2873Ni; **C.** NL-LucR.2873Nipd; and **D.** NL-LucR.1157ipEL. SHIVIG (red solid and green dashed lines) or nRM IgG (grey solid and dashed lines) were assayed in presence of 10% fresh (solid lines) or heat-inactivated (dashed lines) normal human serum as a source of complement. Each data point represents the mean ± SEM (n = 3). All experiments were repeated at least twice.

Next, we tested the number of transmitted virus variants (Figure 5A), using plasma samples collected at peak viremia. We amplified a 570 bp *env* fragment spanning the V1/V2 region. For the final single-genome analysis (SGA), we obtained and sequenced ≥10 individual clones per RM as well as 20 for the SHIV-2873Nip stock. Five quasispecies were observed in the virus stock, whereas control macaques demonstrated a median of two variants. The median number of quasispecies was 2.5 for Group 1 (400 mg/kg) and only one variant for Group 2 (675 mg/kg). In contrast, in Group 3 macaques (25 mg/kg), the number of variants ranged from two to six with a median of three variants, which was significantly different from the median number of quasispecies observed for the control group (*P* = 0.032 by Mann–Whitney test with Holm correction for multiple comparisons). These results imply increased virus acquisition at the low SHIVIG dose.

### SHIVIG at low concentration enhances virus infection in the presence of complement in vitro

The surprise finding of an increased number of transmitted SHIV-2873Nip variants prompted us to postulate infection-enhancing activity resulting from low SHIVIG doses. To test this possibility, the SHIVIG preparation was tested for C’-ADE activity in the CD21^+^ SupT1.R5 cell line using two viruses containing envelopes closely related to the challenge virus, NL-LucR.2873Ni and NL-LucR.2873Nipd. NL-LucR.2873Ni bears the envelope derived from SHIV-2873Ni, which is the parental virus used to obtain SHIV-2873Nip, the challenge virus, through passaging in RMs. NL-LucR.2873Nipd carries Env from SHIV-2873Nipd, which was isolated from an animal that developed AIDS and thus represents a late form of the same virus. SHIVIG enhanced infection of both viruses when fresh normal human serum was present as a source of complement (Figure 5B-C). Infection caused by “early” NL-LucR.2873Ni virus was enhanced by almost 3-fold, while infection by “late” NL-LucR.2873Nipd was augmented up to 16-fold. No significant enhancement was seen when the normal serum was heat-inactivated to destroy complement. Control IgG isolated from a naïve RM (nRM) did not show any enhancement effect. While SHIVIG at 1.27 mg/ml showed 50% neutralization of NL-LucR.2873Ni in the presence of fresh serum, NL-LucR.2873Nipd could not be neutralized. To control whether neutralization could be achieved in this assay, we tested the tier 1 NL-LucR.1157ipEL that carries an Env closely related to viruses against which SHIVIG had been raised (Additional file 1: Table S1). As shown in Figure 5D, SHIVIG completely neutralized the tier 1 NL-LucR.1157ipEL in the presence of either fresh or heat-inactivated complement. Two clade C viruses (Ce1086 and Du151) from acute, sexually acquired infections and heterologous to challenge virus were also tested. SHIVIG enhanced the infection (up to 6-fold) of both viruses at lower concentrations and neutralized them at higher concentrations when fresh normal human serum was present as a source of complement (data not shown).

### Do SHIVIG concentrations yielding maximal C’-ADE in vitro correlate with SHIVIG plasma levels of Group 3 RMs given 25 mg/kg?

The C’-ADE data prompted us to compare SHIVIG concentrations that gave the highest degree of enhancement in the in vitro assay with SHIVIG plasma levels in Group 3 RMs. Among treated macaques, SHIVIG plasma concentrations on day 1 and day 8 after the administration varied between 180 to 250 μg/ml and 70–130 μg/ml, respectively. The peak of enhancement of infection with NL-LucR.2873Ni in C’-ADE assay was observed in the same range of SHIVIG concentrations, 15.7 to 141 μg/ml. This finding strongly suggests an association between the in vitro observed C’-ADE of infection and increased virus acquisition in vivo.

## Discussion

Passive immunization with polyclonal SHIVIG given to RMs that were subsequently exposed repeatedly to low doses of the pathogenic R5 SHIV-2873Nip yielded partial protection at 400 mg/kg as evidenced by statistically significant lower peak viral RNA loads compared with control RMs. The SGA data suggest the possibility of increased viral acquisition at the low SHIVIG dose of 25 mg/kg. When tested ex vivo in the presence of fresh complement, low SHIVIG concentrations showed significant C’-ADE when tested with viruses carrying envelopes related to the challenge virus or other HIV-Cs. These results suggest the possibility that the polyclonal SHIVIG contained Abs with the ability to either partially protect or to facilitate virus acquisition.

These surprising data were generated in a primate model that sought to replicate as many aspects of HIV-1 transmission among humans as possible. Specifically, we chose to perform an upfront heterologous challenge, in contrast to earlier studies that matched the IgG preparation with the challenge virus Env [9,10,19,20]. We also elected to perform multiple low-dose challenges instead of using a single high-dose of virus. The low-dose challenge regimen resulted in a low number of transmitted viral variants in our control animals, a situation similar to that observed in humans. Lastly, the exclusive R5 tropism of our challenge virus reflects that of acutely transmitted founder viruses isolated from humans.

Ab-mediated enhancement of HIV-1 acquisition has been implicated in a recently published subgroup analysis of the Vax004 AIDS vaccine efficacy trial that involved gp120 immunogens [21]. This study linked Fcγ receptor IIIa (FcγRIIIa) genotype with a significantly increased HIV-1 acquisition rate for vaccinees with low behavioral risk of infection and homozygosity for the FcγRIIIa V allele. Other evidence for in vivo increased viral acquisition following immunization stems from experiments involving SIV [22], feline immunodeficiency virus [23-25], and equine infectious anemia virus [26]. Enhancement of viral acquisition and/or higher viral loads or earlier viremia by pre-existing Abs are well-known phenomena for dengue virus [27,28], Murray Valley encephalitis virus [29], respiratory syncytial virus [30], Ebola virus [31] and measles virus [32].

Another recent report described a passive immunization study conducted in HIV-infected pregnant women and their infants in Uganda with the aim of testing anti-HIV-1 Ig (termed HIVIGLOB) for its ability to lower the risk of mother-to-infant virus transmission [33]. Two groups of pregnant women were enrolled; all mothers and their infants were treated with single-dose nevirapine (NVP) according to standard local protocol. Half of the women and their children also received HIVIGLOB (maternal dose, 200 mg/kg at week 36–38 of gestation). The infants received HIVIGLOB at 400 mg/kg within 18 h of delivery. At birth, 9.1% of infants born to HIVIGLOB-treated mothers were HIV positive compared with 4.1% of control infants; the difference was statistically significant. The higher HIV-1 infection rate of HIVIGLOB-treated infants persisted throughout six months of follow-up, although differences at later time points were not statistically significant. In essence, passive immunization with HIVIGLOB did not prevent HIV-1 acquisition in any infants born to infected mothers, and may have enhanced in utero HIV-1 transmission.

Ab-mediated increased viral acquisition was also suggested in a recent study of Burton et al. [34], who performed passive immunization with human monoclonal Abs (mAbs), including the anti-CD4 binding site mAb b6, which is weakly neutralizing. The latter provided no protection from virus acquisition when tested against intravaginal challenge with SHIV_SF162P4_ (tier 1) or SHIV_SF162P3_ (tier 2). SGA revealed a significantly higher number of newly transmitted quasispecies among the b6-treated monkeys compared with the control groups, which is compatible with increased virus acquisition.

Ab-mediated enhancement of lentiviral infection can occur through different mechanisms. The VAX004 data implied that increased risks of HIV-1 acquisition occurred through FcγR-mediated enhancement. Such a mechanism was first described by Takeda and Ennis [35]. Their studies involving cultured monocytes/macrophages demonstrated IgG-linked enhancement of infection only in the presence of surface-expressed Fc-receptors (FcRs) and the IgG constant region. This enhancement still required virus entry through CD4, implying that FcR-bearing cells may enhance infectivity *in trans*. The second major mechanism was first described by Robinson et al., who demonstrated the critical role of complement in C’-ADE [36]. This activity was found to be highly prevalent in individuals with acute HIV-1 infection who had developed binding Abs but no autologous nAbs yet; strikingly, enhancement of infection reached levels up to 350-fold and was not only seen with autologous virus, but also with different virus isolates [37].

Prompted by our observation of an increased number of quasispecies seen in RMs given the low SHIVIG dose, we examined this preparation for C’-ADE activity in vitro. Significant enhancement of infection was seen at low SHIVIG concentrations, whereas higher concentrations of SHIVIG showed some virus-inhibitory activity, thus providing a potential mechanism for the increased number of quasispecies seen in the RMs treated with the low-dose SHIVIG. The complex interactions of HIV-1 with complement, including enhancement of infection, have been reviewed [38]. In this context, it is worth mentioning the recent suboptimal outcome of the phase IIb HVTN 505 trial [39]. The multicomponent immunogens contained envelope and possibly may have induced low levels of anti-Env Abs.

One of our initial goals for the passive immunization using the tier 2 SHIV-2873Nip had been to compare in vivo protection with in vitro neutralization titers of serum samples collected at the time of virus exposure. We now realize that the role of Abs directed against HIV-1/SHIV is more complex in vivo and that the currently used neutralization assays have a narrow focus on prevention of virus entry. A more complex interaction of Abs and virus with primary cells is captured to a certain degree in PBMC-based assays but only if the Ab-virus mixture is left with the cells for several days, a protocol we have followed when assessing SHIVIG in vitro. In contrast, most neutralization assays include a washing step, in which the virus-Ab mixture is removed, thus not allowing ADCC activity to impact the final readout. Currently, routine neutralization assays do not query the influence of complement, and their lack of predictive value may be due to their oversimplified readout and lack of probing mechanisms that may influence the outcome in vivo.

To summarize, passive immunization with SHIVIG yielded partial protection at higher doses and may have increased viral acquisition at the low dose – a perplexing finding. Future studies are required to address the following questions: Can the Ab-mediated infection-enhancing activity be separated from protective functions, such as neutralization, ADCVI and ADCC? More important still, can immunogens be designed that will elicit protective but not infection-enhancing Abs? Will it be possible to induce durable nAb responses at sufficiently high levels to counteract any potential Ab-mediated enhancement of infection?

## Conclusions

SHIVIG displayed the key characteristics of human Abs raised upon HIV-1 infection in humans. Passive immunization with SHIVIG yielded partial protection at higher doses and evidence of increased acquisition at the low dose. Our data imply that the polyclonal SHIVIG preparation contained Abs with the ability to either protect the host or to facilitate viral acquisition. These results were generated in a primate model that replicates as many aspects of HIV-1 transmission among humans as possible, including R5 tropism and a tier 2 neutralization phenotype of the challenge virus as well low-dose repeated mucosal challenges with heterologous virus.

## Methods

### Animals

RMs were housed at the Yerkes National Primate Research Center (YNPRC, Atlanta, GA) in accordance with standards of the National Institutes of Health Guide for the Care and Use of Laboratory Animals. Animal experiments were approved by the Institutional Animal Care and Use Committees at Emory University and the Dana-Farber Cancer Institute (DFCI) via a Collaborating Institution Animal Use Agreement. Blood was collected under ketamine or Telazol anesthesia.

### Proteins and peptides

HIV-1 Tat, HIV-1_UG37_ gp140 (clade A), HIV-1_BaL_ gp120 and HIV-1_IIIB_ gp120 (clade B), HIV-1_CN54_ gp120 and HIV-1_96ZM651_ gp120 (clade C) and HIV-1_UG21_ gp140 (clade D) along with consensus clade C peptides were kindly provided by the NIH AIDS Research and Reference Reagent Program. SHIV-1157ip gp120 and gp160 were kindly provided by Dr. S.-L. Hu. SIV Gag was from Immuno Diagnostics Inc.

### SHIVIG preparation

Total IgG was isolated from sera of RMs infected with SHIV-1157ip [12], SHIV-1157ipd3N4 [15], or related viruses as published [10]. Heat-inactivated RM sera were diluted with PBS, IgG was isolated by chromatography (Protein G Sepharose, GE Healthcare) followed by buffer exchange to PBS, and concentrated by Amicon ultrafiltration (50 kDa cut-off membrane, Millipore). All IgG preparations from individual RMs were analyzed for neutralizing activity and then combined, concentrated to 26.2 mg/ml, filter-sterilized and tested for the presence of endotoxin; all preparations contained <0.02 EU per mg of IgG.

### SHIVIG administration

SHIVIG was administered intravenously every two weeks, 24 h before the first, third and fifth low-dose viral challenges. Group 1 RMs received SHIVIG at 400 mg/kg, Group 2 at 675 mg/kg, Group 3 at 25 mg/kg, and control Group 4 macaques were left untreated. A maximum of three SHIVIG administrations was given. SHIVIG infusions were stopped once an animal had >10^4^ vRNA copies/ml.

### ELISAs

ELISA plates (Nunc) were coated with 1 μg/ml of HIV proteins in carbonate buffer, pH 9.6. After washing, plates were blocked with 2% BSA (Sigma-Aldrich), 0.05% Tween-PBS (blocking buffer). Plates were then incubated with serial dilutions of SHIVIG in triplicates. After washing, plates were developed by incubation for 1 h with rabbit anti-monkey IgG HRP-conjugated Ab (Sigma) and by adding 100 μl of *o*-phenylenediamine solution.

For competitive ELISA with bnmAbs b12, VRC01 and 4E10, plates were coated with 0.1 μg/ml of HIV-1_CN54_ gp120. After washing and blocking with blocking buffer, plates were incubated with b12, VRC01 or 4E10 (0.5 μg/ml) along with different concentrations of SHIVIG in duplicates. After extensive washing, plates were incubated for 1 h with biotinylated goat anti-human IgG (RM IgG adsorbed; Southern Biotech) and 1 h with HRP-streptavidin (Jackson Immunoresearch). Plates were again washed, developed with 3,3′,5,5′-tetramethylbenzidine solution (TMB; Invitrogen).

Competitive ELISA with mAb 17b was performed as described [40]. In brief, plates were coated with 1 μg/ml of sheep anti-gp120 Ab (Aalto Bio Reagents Ltd.) and blocked with blocking buffer. HIV-1_CN54_ gp120 (1 μg/ml) was incubated with soluble CD4 (40 μg/ml) for 45 minutes. Then, mixture was diluted 20-fold by blocking buffer, added to plates and incubated for 1.5 h. After washing bnmAb 17b (0.5 μg/ml) along with different concentration of SHIVIG was added to the plate. Binding was detected as described above.

ELISA with consensus clade C peptides was performed essentially as described above. Plates were coated with pools of 5 peptides (5 μg/ml for each) in triplicates, blocked and probed with 5 μg/ml of SHIVIG. To detect binding, plates were incubated with anti-monkey IgG HRP-conjugated Ab and developed with TMB solution.

### Determination of SHIVIG and total IgG concentrations in RM samples

The concentration of SHIVIG in plasma samples was determined by ELISA. The 96-well plates were coated with gp120 of HIV-1_BaL_ at 0.6 μg/ml for plasma SHIVIG determination and 1 μg/ml for measurement of SHIVIG content in vaginal lavages. Plates were incubated overnight at 4°C. After blocking and washing, serially diluted, heat-inactivated plasma samples were added to plates in triplicates. Vaginal lavage samples were thawed, diluted 1:1 with blocking buffer, heat-inactivated and added to plates in triplicate. SHIVIG was included as a standard ranging from 0.156 to 1 μg/ml. To detect binding, plates were incubated with rabbit anti-monkey IgG HRP-conjugated Ab and developed with One Step Ultra TMB Substrate (Thermo Fisher Scientific). To determine the half-life of SHIVIG, natural logs of SHIVIG plasma levels were plotted as a function of time from the end of infusion. Slopes of the linear graphs were determined by least-squares analysis. Half-lives were calculated as t_1/2_ = −(ln 2)/m.

Total IgG content in vaginal lavage fluids was assessed by ELISA. Briefly, plates were coated with rabbit anti-monkey IgG-whole molecule (Sigma), blocked and probed with serially diluted vaginal lavage samples in triplicates. After washing, bound IgG was detected with rabbit anti-monkey IgG HRP-conjugated Ab and developed with One Step Ultra TMB Substrate. Naïve RM (nRM) IgG served as a standard.

### In vitro neutralization assays

The TZM-bl assay was performed as described [41]. In brief, virus was added to cells in the presence of DEAE-dextran (Sigma), washed 1× on day 1 and luminescence was measured on day 2 using luciferase substrate Bright-Glo (Promega).

Human PBMC-based assay was performed as described [42]. Serially diluted SHIVIG was incubated with virus for 1 h at 37°C. The virus/SHIVIG mixture was then added to the cells. Supernatant aliquots were harvested every other day starting on day 3. To remove anti-Gag antibodies that could interfere with the p27 assay read-out, plates were washed 5 times on day 4. The levels of p27 in supernatants were assayed first in wells containing only cells plus virus. When p27 levels were in the linear phase of increase in these control wells, neutralization was assessed for test samples. To analyze the role of NK cells, the same PBMC assay was run with and without NK cells from the same donor. PBMC were depleted of NK cells using an anti-CD56 mAb linked to magnetic beads according to the manufacturer’s protocol (Stemcell Technologies).

### Ab-dependent cell-mediated viral inhibition (ADCVI) assay

IgG ADCVI activity was measured as described [43]. Briefly, SHIV-2873Nip-infected CEM.NKr.CCR5 target cells were incubated with SHIVIG or HIVIG (IgG from a pool of HIV-positive donors) and with fresh PBMC effector cells from normal human donors (effector-to-target cell [E:T] ratio of 10:1). Cells were washed to remove Ab on day 4. On day 7, supernatants were assayed for p27 by ELISA. IgG from naïve RM and IVIG (IgG from a pool of healthy donors) were used as negative controls. Percent virus inhibition was calculated with regards to the value obtained with the negative control used at the same concentration as described [43]. All samples were assayed in triplicates.

### Ab-dependent cell-mediated cytotoxicity (ADCC) assay

Measurement of ADCC activity was performed as described [44]. Briefly, target CEM-NKr cells were coated with HIV-1_96ZM651_ gp120 clade C. Human PBMC served as effectors and were used at an effector to target (E:T) ratio of 50:1. ADCC titers are defined as the reciprocal dilution at which the % killing was greater than the mean % killing of the negative controls plus three standard deviations. SHIVIG-derived Fab-fragments were used as a negative control.

### Complement-mediated Ab-dependent enhancement (C’-ADE) assay

Viruses used were all clade C. Env genes from SHIV-2873Ni, SHIV-2873Nipd and SHIV-1157ipEL were cloned into the pNL-LucR.T2A vector containing *Renilla* luciferase gene inserted into pNL4-3 DNA [45]. Virus stocks were produced in 293 T cells (NL-LucR-2873Ni, NL-LucR-2873Nipd and NL-LucR-1157ipEL) or human PBMC (HIV-C strains Ce1086 and Du151). C’-ADE of virus infection was measured in SupT1.R5 cells as described [37]. Virus was incubated with serial dilutions of SHIVIG or nRM IgG in duplicates in the presence of 10% fresh human serum as source of complement (Sigma) for 1 h at 37°C. As control, SHIVIG was also assayed in the presence of 10% human serum heat-inactivated (56°C, 1 h) to destroy complement activity. Percent neutralization was determined by calculating the difference in average relative luminescence units (RLU) between test wells (cells + serum + virus) and cell control wells (cells only), dividing this result by the difference in average RLU between virus control (cell + virus) and cell-control wells, subtracting from 1 and multiplying by 100. Negative values are indicative of infection-enhancement.

### Single-genome analysis (SGA)

The 570 base-pair *env* fragment spanning V1/V2 was amplified as described [46]. In brief, total RNA was extracted and purified from RM plasma using QIAamp viral RNA kit (Qiagen, Valencia, CA). Reverse transcription was performed using the Superscript III kit (Invitrogen, Carlsbad, CA) according to the manufacturer’s instructions using SHIV-2873Nip *env*-specific primers. The cDNA was serially diluted and dispersed in 96-well plates to identify a dilution constituting <30% of the total number of PCR-positive wells as described [47]. Nested PCR was performed on the cDNA to amplify the 570 bp *env* fragment. The following primers were used; for the first round forward primer (F): 5′-ATGAGAGTGAAGGAGAAATATCAGCACTTTGTGGAGA-3′ and reverse primer (R): 5′-TTCCTCATCTATATCATCCATATTTTGTTTTCTGTA-3′. For the second round F: 5′-GGTACCTGTGTGGAAAGAAGCAAAAACTACTCTAT-3′ and R: 5′-GGCTACCATTTAACAATAGTTGAGTTGACACCACT-3′. PCR conditions and reagents were used as described [46]. Correctly sized amplicons were identified by agarose gel electrophoresis and sequenced using *env*-specific primers. All sequences were quality checked, analyzed for diversity, viral recombination, PCR and sequencing errors as described [46]. Finally, phylogenetic trees were constructed and the number of variants was estimated.

### Statistical analysis

Mean times to first or peak viremia as well as peak viremia levels were calculated for each SHIVIG group. Distribution assumptions of the outcomes were examined graphically and used to determine the appropriate models for statistical analyses. Generalized linear models were used to compare the outcomes between the control and SHIVIG-recipient groups assuming either a Poisson or negative binomial distribution, as appropriate, with log-transformation. Furthermore, the AUC was calculated for each animal, and group comparisons of log-transformed AUCs were conducted via one-way ANOVA. Adjustments for multiple comparisons were incorporated, as necessary. Additionally, secondary outcomes analyses were conducted within MHC and TRIM5α-resistant genotypes, as numbers allowed. All analyses were conducted using StataMP 11.0 (StataCorp LP, College Station, TX).

## Abbreviations

Ab: Antibody; mAb: monoclonal antibody; nAb: neutralizing antibody; bnmAb: broadly neutralizing monoclonal antibody; ADCC: Antibody-dependent cell-mediated cytotoxicity; ADCVI: Antibody-dependent cell-mediated virus inhibition; AUC: area-under-the-curve; C’-ADE: Complement-dependent antibody-mediated enhancement; Env: Envelope; FcR: Fc-receptor; HIV-C: HIV-1 clade C; MPER: Membrane-proximal external region; IC50: Half maximal inhibitory concentration; i.r.: Intrarectal; RM: Rhesus monkey; SGA: Single-genome analysis; SHIV: Simian Human Immunodeficiency Virus; TCID50: 50% tissue culture infectious dose.

## Competing interests

The authors declare that they have no competing financial interest.

## Authors’ contributions

Contribution: A.M.S., R.A.R., H.C.E. and R.M.R conceived the study and designed the experiments; A.M.S., S N.B., V.S., G.H., S.K.L., J.D.W., H.K.V., S.T., T.B., M.M.M., and J.K.Y. performed the experiments; F.J.N and F.V. managed primate experiments; G.L. and D.N.F. performed ADCVI analysis; I.T. and M.R.-G. performed ADCC assay; V.R.P, M.B. and D.C.M. performed neutralization assay of SHIVIG and RM plasmas; S.R. performed statistical analysis; W.E.J performed TRIM5α genotyping; A.M.S. and R.M.R analyzed the data and wrote the manuscript. All authors read and approved the final manuscript.

## Supplementary Material

Additional file 1: Table S1 Treatment history and clinical parameters for cohort of RMs used to isolate SHIVIG. Click here for file

Additional file 2: Table S2 Genetic characteristics of rhesus monkeys enrolled into the study. Click here for file
