# Supplementary material for: Passive immunization of macaques with polyclonal anti-SHIV IgG against a heterologous tier 2 SHIV: outcome depends on IgG dose
Source: Retrovirology. 2014 Jan 20;11:8. doi: 10.1186/1742-4690-11-8 (PMC3905655; doi:10.1186/1742-4690-11-8)
Supplement: Additional file 1: Table S1 — Treatment history and clinical parameters for cohort of RMs used to isolate SHIVIG. [file 1742-4690-11-8-S1.pdf]

Table S1 Treatment history and clinical parameters for cohort of RMs used to isolate SHIVIG

Monkey	Virus exposure	Other treatments	Viral RNA copies/ml		CD4 count	Number of months infected	Clinical status	Ref.
			Peak	Last				
RAo-8	SHIV-1157ip passage	–	2.8×10^7	<50	624	110	systemic infection, LTNP	[12]
RCt-10	SHIV-1157ipd3N4	DNA prime: pJW-gp160-1157ip pJWSIVgag-pro pJWHIVtat protein boost: SHIV-1157ip gp160 AT-2-inactivated SIV HIV Tat	2×10^7	<50	290	52	sacrificed due to acute pneumonia	unpub. data
RHo-10	SHIV-1157ipd3N4	–	7.1×10^7	250	414	60	persistently viremic	unpub. data
RHy-9	SHIV-1157ip SHIV-1157ipd3N4	DNA prime: pJW-gp160-1048i pJWSIVgag-pro pJWHIVtat protein boost: HIV1084i gp160 SIV Gag-Pol particles HIV Tat	6.3×10^4	<50	694	37	systemic infection, LTNP, sacrificed due to behavior problem	[14]
RJa-9	SHIV-1157ip oral titration	*S. mansoni*	3.7×10^6	<50	596	102	systemic infection, LTNP	[13]
RLu-9	SHIV-1157ip SHIV-1157ipd3N4	HIV1084i gp160 SIV Gag-Pol particles HIV Tat	7.8×10^5	<50	330	122	systemic infection, LTNP	[13]
RMf-9	SHIV-1157ip oral titration	*S. mansoni*	7.2×10^7	<50	479	101	systemic infection, LTNP	[14]
RPo-10	SHIV-1157ip passage	–	5×10^7	2.1×10^5	485	54	sacrificed due to mycobacteriosis	[12]
RTs-7	SHIV-1157ip passage	–	6.6×10^5	3.7×10^4	14	87	systemic infection, death from AIDS	[12]
