# Supplementary material for: Passive immunization of macaques with polyclonal anti-SHIV IgG against a heterologous tier 2 SHIV: outcome depends on IgG dose
Source: Retrovirology. 2014 Jan 20;11:8. doi: 10.1186/1742-4690-11-8 (PMC3905655; doi:10.1186/1742-4690-11-8)
Supplement: Additional file 2: Table S2 — Genetic characteristics of rhesus monkeys enrolled into the study. [file 1742-4690-11-8-S2.pdf]

**Table S2 Genetic characteristics of rhesus monkeys enrolled into the study**

| Group name | RM | TRIM5α genotype | Susceptibility to infection (based on TRIM5α) | MHC genotype[a] |
|---|---|---|---|---|
| | RKg-12 | CYP A / CYP A | less | B17 |
| | RKs-12 | TFP / TFP | less | — |
| Group 1 | RQv-12 | TFP / CYP A | less | — |
| (400 mg/kg) | RRm-12 | TFP / CYP A | less | A01 |
| | RVv-12 | TFP / Q | moderate | B17 |
| | RZn-12 | TFP / Q | moderate | B17 |
| Group 2 | RRs-12 | Q / CYP A | moderate | — |
| (675 mg/kg) | RZu-11 | TFP / Q | moderate | — |
| | RJu-11 | Q / CYP A | moderate | — |
| | RKz-11 | CYP A / CYP A | less | — |
| Group 3 | RNz-11 | Q / CYP A | moderate | — |
| (25 mg/kg) | ROw-11 | Q / Q | most | A01 |
| | RWt-12 | TFP / CYP A | less | — |
| | RZa-12 | CYP A / CYP A | less | B17 |
| | RCa-12 | Q / CYP A | moderate | B17 |
| | REv-12 | TFP / CYP A | less | B08 |
| | RHc-12 | TFP / Q | moderate | — |
| | RHm-11 | Q / CYP A | moderate | A01 |
| | RHv-11 | TFP / CYP A | less | — |
| | RMs-11 | CYP A / CYP A | less | A01 |
| Group 4 | RPl-12 | TFP / Q | moderate | A01 |
| (controls) | RSc-12[b] | TFP / CYP A | less | — |
| | RSm-11 | TFP / TFP | less | A01 |
| | RVu-11 | TFP / TFP | less | — |
| | RWl-11 | TFP / Q | moderate | A01 |
| | RWo-12 | TFP / TFP | less | A01 |
| | RWu-11 | TFP / CYP A | less | — |
| | RZw-10 | TFP / CYP A | less | — |

[a]MHC genotyping done with B17, B08, A01. "—" indicates that the animal was negative for all the 3 genotypes
[b]Monkey RSc-12 had a delay in receiving the 5th weekly i.r. challenge; therefore, only data for this RM including the 4th challenge are included.
